# Myocarditis as a Possible Underlying Cause for Mid-Ventricular Takotsubo Cardiomyopathy: A Case Report

**DOI:** 10.7759/cureus.75813

**Published:** 2024-12-16

**Authors:** Jiana T Baker, Ricardo Cury, Dagmar F Hernandez-Suarez

**Affiliations:** 1 Medicine, Florida International University, Herbert Wertheim College of Medicine, Miami, USA; 2 Radiology, Baptist Health South Florida, Miami, USA

**Keywords:** cardiac mri, cardiomyopathy, ischemia with non-obstructive coronary arteries (inoca), mid-ventricular takotsubo cardiomyopathy, myocarditis

## Abstract

Our case report characterizes a rare presentation of mid-ventricular Takotsubo cardiomyopathy (TTC) in a patient with suspected myocarditis as an underlying cause. Mid-ventricular TTC is a rare variant of TTC presenting with overlapping symptoms and physical exam findings of acute coronary syndrome, which often leads to misdiagnosis as myocardial infarction.

Our case is of a 77-year-old female patient with a history of hyperlipidemia, right breast ductal carcinoma in situ, and diverticular disease who presented to the emergency department for evaluation of chest pain radiating to the jaw with associated nausea and vomiting. She had a similar event eight months earlier and was diagnosed with ischemia with non-obstructive coronary arteries (INOCA) based on cardiac catheterization findings. Family history was notable for myocardial infarction in the patient’s father and paternal grandfather. At presentation, the patient had elevated blood pressure, elevated troponins (initially 1058 ng/L with a repeat level of 11,421 ng/L), and electrocardiogram (ECG) findings of sinus bradycardia without ischemic changes. Cardiac MRI (CMR) revealed sub-epicardial delayed enhancement suggestive of possible myocarditis and diffuse hypokinesis involving the inferolateral left ventricular wall, sparing the basal and apical segments. Left ventricular function was mildly decreased (ejection fraction of 49%) and improved prior to discharge with ejection fraction (55-60%). There were no wall motion abnormalities or significant valve disease.

This case presentation exemplifies a rare manifestation of mid-ventricular TTC that occurred in the setting of underlying myocarditis. Given the patient’s history, elevated troponins, and CMR results suggestive of myocarditis, we hypothesize that underlying myocarditis may have incited the development of mid-ventricular TTC. The absence of identifiable triggers in conjunction with the inferolateral ventricular wall hypokinesis and late gadolinium enhancement on CMR supports our hypothesis. The variability in clinical presentations between the present case and other reported cases of mid-ventricular TTC emphasizes the need for a deeper understanding of this condition, its triggers, and the associated clinical features to reduce the risk of future misdiagnoses. This case report highlights the significance of thorough imaging assessments for patients presenting with INOCA. Further investigations are warranted to determine underlying causes for hemodynamic instability and unique aspects of the mid-ventricular manifestation of TTC, with an emphasis on the potential association of myocarditis as an inciting factor.

## Introduction

Mid-ventricular Takotsubo cardiomyopathy is a rare form of Takotsubo cardiomyopathy (TTC), commonly known as broken-heart syndrome, which is a reversible myocardial injury characterized by regional wall abnormalities in the left ventricle [1]. The classic form is primarily found in postmenopausal females after experiencing significant emotional or physical stressors. Typical TTC is described by the apical ballooning of the left ventricle that occurs during systole and is often associated with basal segment hyperkinesis. Atypical variants include the basal, focal, mid-ventricular, biventricular, isolated right ventricular, and global forms that are characterized based on the region of dysfunction.

Both typical and atypical variants of TTC mimic the clinical presentation of acute coronary syndrome with chest pain, elevated cardiac enzymes, and common electrocardiogram (ECG) changes [1]. Approximately two percent of patients presenting with ACS are diagnosed with TTC and only 14.6% of these cases have the mid-ventricular variant [2,3]. In fact, from 1998 to 2014, just 255 patients were diagnosed with mid-ventricular TTC worldwide [3].

There are several triggers for TTC with apical ballooning including, but not limited to, psychological triggers due to a range of emotions and physical stressors (physical activity, medical conditions, chemical agents, and procedures) [4]. While myocarditis and Takotsubo syndrome were previously believed to be mutually exclusive [5], we are reporting a case where underlying myocarditis seems to have been the trigger for the development of the rare mid-ventricular form of TTC. To our knowledge, this is the first case that describes the clinical course, phenotypic presentation, and imaging results for a patient with mid-ventricular TTC in the setting of possible underlying myocarditis.

## Case presentation

Our case is of a 77-year-old female patient with a past medical history of hyperlipidemia, right breast ductal carcinoma in situ, and diverticular disease who presented to the emergency department (ED) for evaluation of chest pain described as burning and radiating to the jaw and throat with associated nausea and vomiting. She reported that she woke at approximately 04:00 with severe dizziness described as the feeling of the room spinning, rendering her unable to ambulate to the bathroom independently. This feeling persisted upon presentation to the hospital. The symptoms continued to increase in severity until the patient developed nausea resulting in 12-13 bouts of emesis. Despite these multiple episodes leaving her with very little gastric content, a 10/10 burning pain of the chest, along with neck and throat pain persisted. Her concern prompted her to seek care at her local ED two hours later. 

The patient stated that she had previously suffered similar symptoms and sought care at a different healthcare facility about eight months prior to the current presentation. She was initially diagnosed with a non-ST elevation myocardial infarction (NSTEMI) and underwent coronary angiography with ventriculogram and left heart catheterization. The results revealed non-obstructive coronary artery disease as well as normal left ventricle systolic function and filling pressures. Thus conservative management was recommended. The patient was diagnosed with ischemia with non-obstructive coronary arteries (INOCA) and started on rosuvastatin by her primary care provider. There was no further investigation into the underlying cause. 

At baseline, the patient reported being independent in all the activities of daily living, regular exercise with no anginal equivalent, and no previous episodes of volume overload requiring diuretic therapy. She had no history of tobacco, alcohol, or illicit drug use. She reported a family history of myocardial infarction in both her father and paternal grandfather. The only remarkable finding from the physical exam was an elevated blood pressure of 153/72 mmHg (reference value <120/80 mmHg). She did not appear in acute distress and had a regular heart rate and rhythm. There was no notable pedal edema, jugular venous pulsations, skin pallor, headache, or shortness of breath. Her lungs were clear on auscultation and there was no evidence of abnormal carotid upstrokes. The Dix-Hallpike maneuver was attempted to identify benign paroxysmal positional vertigo which did not elicit a response. The patient's lab results revealed a few significant findings. While she was afebrile, she had minor leukocytosis with a white blood cell count that was mildly elevated at 12.3 K/µL (reference range 4.5-11 K/µL). Her chemistry profile demonstrated normal sodium levels and hypokalemia with a potassium level of 3.3 mmol/L (reference range 3.6-5.2 mmol/L), for which she was given 40 mEq of potassium orally. Most notably, the patient's initial troponin was slightly elevated at 1058 ng/L but increased significantly to 11,421 ng/L on repeat testing (reference value <14 ng/L). Creatinine, glomerular filtration rate, and blood urea nitrogen were all within normal range. Additionally, her blood glucose level was elevated at 151 mg/dL (reference value <100 mg/dL), and she was found to have hypocapnia with a CO_2_ level of 20 mmol/L (reference range 23-29 mmol/L).

**Figure 1:**
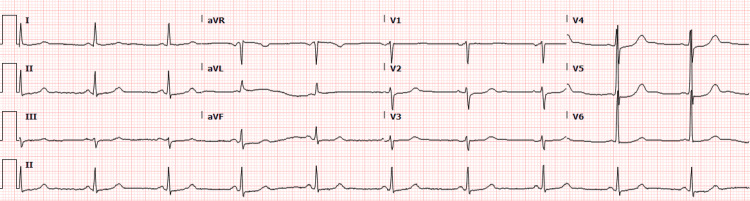
ECG on presentation reflecting sinus bradycardia without ischemic changes

Initial imaging workup was performed with a chest X-ray that revealed no evidence of acute intrathoracic pathology. Serial ECGs displayed sinus bradycardia (ventricular rate of 50 beats per minute) without ischemic changes (Figure 1). The patient was treated with heparin, aspirin, morphine, nitroglycerin as well as antiemetics. Given the patient’s symptoms, physical exam findings, laboratory tests, and imaging, acute coronary syndrome/NSTEMI was considered. Hence, the patient was transferred to a higher level of care for interventional cardiology evaluation and possible invasive coronary risk stratification. 

**Figure 2:**
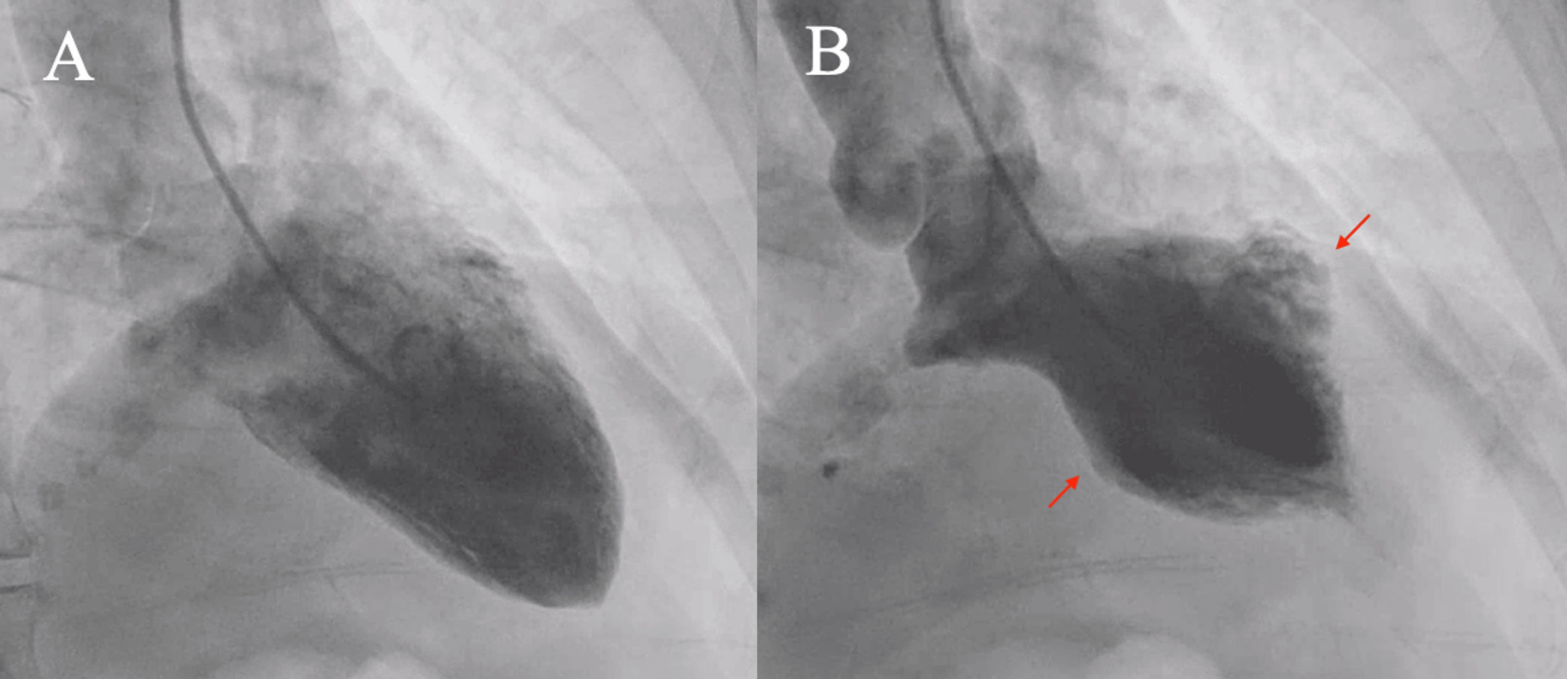
Left ventriculogram of right anterior oblique orientation in diastole (A) and systole showing hypo/dyskinesis of the midventricular section (arrows) with a hyperdynamic base and apex (B)

After being transferred and admitted to the inpatient adult stepdown unit, the patient had a cardiology consultation. She was continued on medications relevant to the acute coronary syndrome protocol (including heparin, aspirin, clopidogrel, nitroglycerin and morphine) with the exception of a beta-blocker due to baseline bradycardia. She was now thought to be experiencing coronary artery vasospasm based on her ECG results and the lack of epicardial obstructive disease during her prior coronary angiography. The patient underwent urgent cardiac catheterization which revealed minimal findings of luminal irregularities in the left anterior descending and left circumflex arteries and hypo/dyskinesis of the ventricular segment with hyperdynamic basal and apical segments seen on ventriculogram (Figure 2). 

Echocardiography demonstrated concentric left ventricle remodeling and aortic sclerosis characterized by thickening and calcification of aortic leaflets without ventricular outflow obstruction, consistent with age-related degenerative changes. Regurgitation was detected across the aortic, pulmonic, and tricuspid valves, which was likely a consequence of the wall motion abnormalities. Additionally, the right ventricular systolic pressure was elevated (40-50 mmHg) possibly resulting from the tricuspid valve regurgitation.

**Figure 3:**
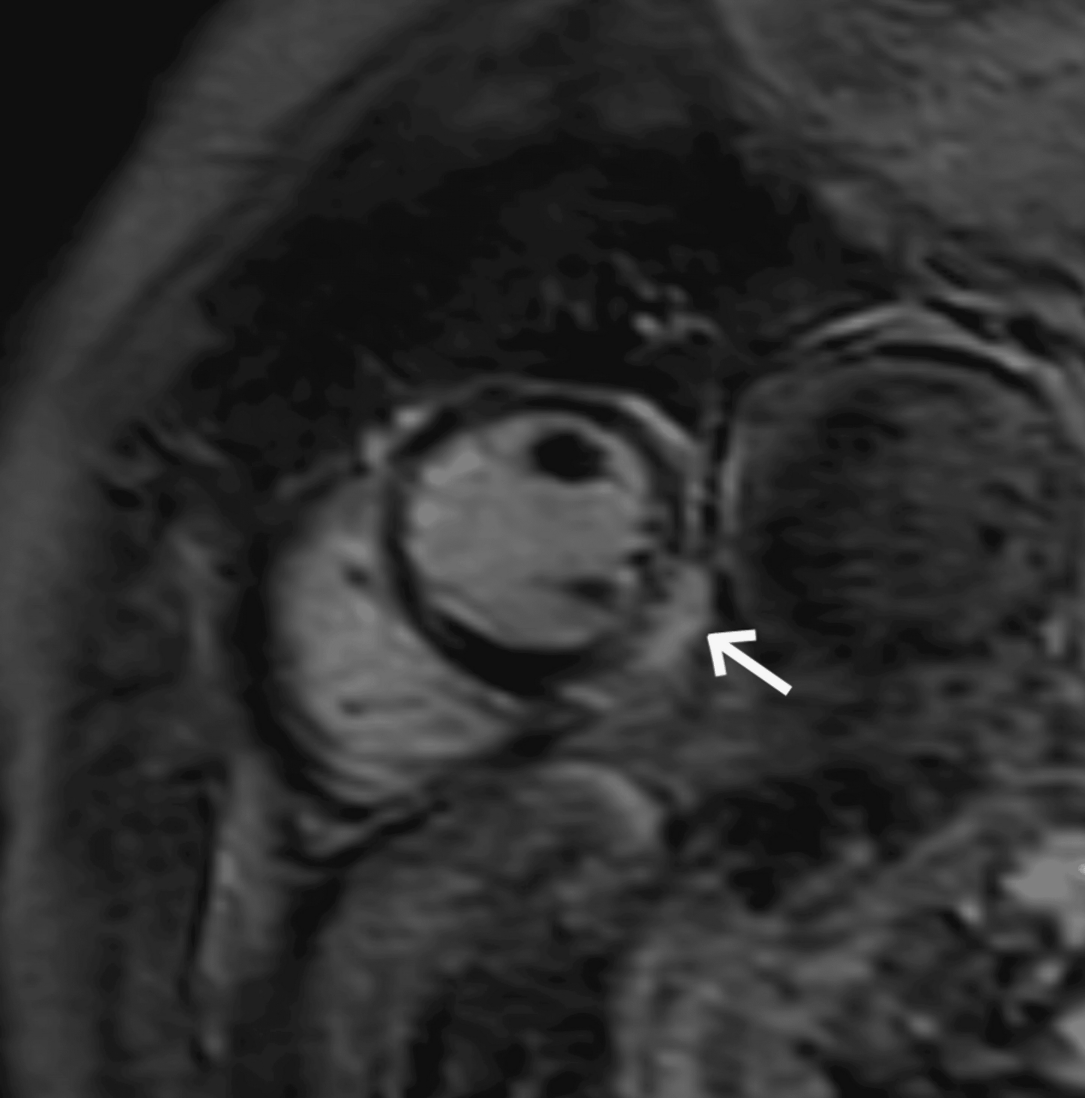
Delayed-enhanced MRI 10 minutes after gadolinium administration demonstrating sub-epicardial delayed enhancement (arrow) in the mid-inferolateral wall suggesting associated myocarditis

Given the decreased systolic function (ejection fraction 49%) noticed in both the ventriculogram and ECG, further morphological assessment was performed with a cardiac MRI (CMR). The imaging demonstrated small sub-epicardial delayed hyperenhancement involving the inferolateral wall that was suggestive of possible myocarditis (Figure 3).

There was also diffuse hypokinesis involving all the mid-left ventricular walls with sparing of the basal and apical segments which can be seen in an atypical form of stress-induced cardiomyopathy/TTC (Video 1 and Video 2).

**Video 1:** Cine 2-chamber view demonstrating severe hypokinesis in end-systole of the anterior and inferior walls only at the mid-ventricular level

**Video 2:** Cine 4-chamber view demonstrating severe hypokinesis in end-systole of the septal and lateral walls only at the mid-ventricular level

Global left ventricular function was mildly decreased with an estimated ejection fraction of 49%. The right ventricle had normal structure and function without wall motion abnormalities and an ejection fraction of 72%. Additionally, the patient had mild aortic and tricuspid valve regurgitations with small bilateral pleural effusions. Overall, the patient’s presentation was consistent with transient mid-ventricular ballooning syndrome which is an atypical variant of stress-induced cardiomyopathy. Given the patient's resolution of symptoms and minimal findings on cardiac catheterization, she was later discharged and instructed to follow-up as an outpatient with the cardiology department.

## Discussion

Mid-ventricular TTC is extremely rare amongst patients presenting with symptoms classically associated with acute coronary syndrome and elevated troponins. It is unique because it can occur without ECG changes, while other variants will likely present with ST-segment elevation in the anterior precordial leads as well as ST-segment depression, QT prolongation, T-wave inversion, and abnormal Q waves [1]. Our patient had no ischemic changes in the ECG during the initial presentation. Other cases of mid-ventricular TTC have noted subtle ST-elevations in leads V1 and V2 as well as ST-T changes corresponding to the lateral and inferior walls after experiencing an emotional stressor [6]. Demirelli et al. (2015) described how a patient experienced an emotional stressor after learning of the passing of her son and exhibited normal sinus rhythm with T-wave inversion on leads V1-V4 [7]. Giannattasio et al. (2021) reported dynamic T-wave inversion sparing apical leads V3-V4 in another case, which was described as “Wellen’s pattern” [8]. This variation and ECG changes reinforce the need to better understand the differences in TTC presentation based on the initial triggers.

The assessment of morphological functioning is essential to diagnose TTC due to overlapping symptoms with acute coronary syndrome and the non-specific ECG changes that occur in the different forms of TTC. Our patient initially had a coronary angiogram eight months before her presentation without significant findings. However, she did not undergo further imaging assessment with a ventriculography, echocardiogram, or cardiac CT/CMR which would have likely shown the wall motion abnormality that was detected on the second presentation. Nikias et al. (2022) reported a patient with new-onset heart failure, elevated troponins, and normal ECG results, who demonstrated new left ventricular wall motion abnormalities on transthoracic echocardiogram that resolved on re-examination three weeks later [9]. Both cases illustrate the significance of echocardiography and other cardiac morphological functioning tests when assessing patients experiencing acute hemodynamic instability to identify changes that would otherwise not be detected on either ECG or coronary angiography.

The CMR performed with gadolinium contrast during the patient’s second presentation also detected several deviations in morphological functioning. The patient was found to have severe hypokinesis of the mid-left ventricular walls with preserved regional wall motion at the base and apical segments consistent with our diagnosis of mid-ventricular TTC. This was associated with a reduced left ventricular ejection fraction of 49% and bilateral pleural effusions as well as mild aortic and tricuspid valve regurgitations. When compared to the other forms of TTC, Ojha et al. argued that the mid-ventricular form has a greater degree of left ventricular dysfunction and hemodynamic compromise, consistent with the dysfunction seen in our case [10]. Acute mitral regurgitation has been noted to be more common in the apical type of TTC due to the systolic anterior motion of the mitral leaflet and associated left ventricular outflow tract obstruction [11]. Mitral regurgitation occurs in around 15-21.5% of TTC cases [12-14]. However, the frequency for cases of mid-ventricular TTC is not known. A previous case of mid-ventricular TTC noted mitral regurgitation that was independent of left ventricular outflow obstruction and occurred amidst the transition of the cardiomyopathy to the apical form of the condition [15]. Our patient experienced left ventricular dysfunction along with aortic and tricuspid valve regurgitation without transitioning to another form of TTC prompting the question of whether valvular dysfunction is also associated with the mid-ventricular TTC. 

The classic form of TTC has been found to be associated with an array of emotional (including psychologic stressors, trauma, and other experiences provoking strong emotion) and physical (medical conditions, injuries, surgery, anesthesia, etc.) triggers [4]. While there are no known triggers for mid-ventricular TTC, case reports have noted its potential association with assorted emotional and physical triggers [6,7,16,17]. Initially, our patient’s condition appeared to occur in the absence of any apparent triggers, however, we now believe that underlying myocarditis may have triggered the development of mid-ventricular TTC. Previously, myocarditis and TTC were considered to be mutually exclusive [5,18]. The absence of late gadolinium enhancement on CMR results was requirement for a diagnosis of TTC. However, a study using gadolinium-enhanced global and regional CMR analysis in patients with left ventricular apical ballooning syndrome found myocarditis as a possible etiology [19]. Our findings demonstrated a small sub-epicardial delayed hyperenhancement involving the mid-inferolateral wall that was suggestive of possible myocarditis. Similar to our patient, the majority of the patients in the previously-mentioned study had leukocytosis, however, many also had a preceding febrile illness that was not seen in our case [19]. Given the absence of a preceding illness, it is possible that the delayed hyperenhancement may be characteristic of this rare form of TTC and not seen in the other variations. Since there is minimal information available currently regarding the clinical conditions associated with mid-ventricular TTC in the absence of apparent triggers, our findings suggest that myocarditis could be, in theory, associated with this TTC variant.

## Conclusions

This report describes a case of mid-ventricular TTC that occurred in the absence of any identifiable triggers and characterizes the potential clinical conditions and deviations in cardiac morphological functioning that may be associated with this variation of TTC. Since there is little evidence supporting the occurrence of mid-ventricular TTC in the absence of any emotional or physical triggers, we hypothesize that the underlying myocarditis may have incited the development of mid-ventricular TTC in our patient based on the result of the late gadolinium enhancement on CMR. The variability between our case and other reported cases of mid-ventricular TTC demonstrates the need for a better understanding of the condition, its triggers, and clinical presentation to avoid future misdiagnoses. Further research is warranted to determine the underlying causes for the hemodynamic instability as well as aspects that are unique to the mid-ventricular form of TTC and whether myocarditis plays a critical role as a possible trigger.
